# Toll-like receptor 2 induces adenosine receptor A2a and promotes human squamous carcinoma cell growth via extracellular signal regulated kinases ½

**DOI:** 10.18632/oncotarget.23784

**Published:** 2017-12-30

**Authors:** Chithra Devi Palani, Lalitha Ramanathapuram, Aroonwan Lam-ubol, Zoya B. Kurago

**Affiliations:** ^1^ Dental College of Georgia, Augusta University, Augusta, GA, USA; ^2^ Memorial Sloan Kettering Cancer Center, New York, NY, USA; ^3^ Faculty of Dentistry Srinakharinwirot University, Wattana, Bangkok, Thailand; ^4^ Medical College of Georgia, Augusta University, Augusta, GA, USA

**Keywords:** oral cancer, squamous cell carcinoma (SCC), toll-like receptor (TLR)2, adenosine receptors (AR), MAPK

## Abstract

Patient treatment for oral squamous cell carcinoma (OSCC) not associated with *Human papillomavirus* remains problematic. OSCC microenvironment is typically inflamed and colonized by microorganisms, providing ligands for toll-like receptors (TLR). In immune cells TLR2 and TLR4 activate NF-kB and extracellular signal regulated kinase (ERK)1/2 pathways, leading to upregulation of inhibitory adenosine receptors A2a and A2b, and reduction in stimulatory A1 and A3. How TLR and adenosine receptors function in SCC cells is not understood. To address this gap, we evaluated TLR and adenosine receptor expression and function in human OSCC cells and keratinocytes. TLR2 and A2a were co-expressed in pre-cancer and SCC cells of 17 oral specimens. *In vitro*, 5/6 OSCC lines expressed more TLR2 than TLR1, 4 or 6 mRNA. TLR2 ligands stimulated A2a expression in TLR2-high cell lines, but no A1 or A3 was detected with or without stimuli. In TLR2-high OSCC, TLR2/1, 2/6 and adenosine receptor agonists activated ERK1/2. TLR2-mediated ERK1/2 phosphorylation resulted in accumulation of c-FOS, ERK-dependent cell proliferation and reduced caspase-3 activity. Similar ERK1/2-dependent proliferation and decreased caspase-3 activity were caused by combined TLR2 and adenosine receptor stimuli. We conclude that TLR2 and adenosine receptor agonists, known to be present in the tumor microenvironment, may contribute to OSCC progression in part via direct effects on the ERK1/2 pathway in squamous carcinoma cells.

## INTRODUCTION

Squamous cell carcinoma (SCC) is a tenacious and aggressive cancer of epithelial cells that occurs in many organs, and is especially prevalent at mucosal and skin surfaces. In the oral cavity, SCC represents 85–90% of all malignancies [1]. Here we focus on the major group of Human papillomavirus (HPV)-negative oral (O)SCC, which represent >90% of oral cancer [2]. Frequently, OSCC develop via pre-cancerous changes called epithelial dysplasia, which is recognized due to the characteristic morphologic abnormalities confined to some or most of the mucosal squamous epithelial cell layers [3]. Notably, in contrast to most solid cancers, the level of cell differentiation has little impact on the overall survival of patients with OSCC [3], suggesting that factors outside the malignant cells are important. OSCC develop in the context of surface-associated microbes, and several studies have identified various commensal and pathogenic Gram-positive (G^pos^) and Gram-negative (G^neg^) bacteria that colonized these cancers [4–9], while OSCC cells were deficient in the production of several anti-microbial peptides [10]. So far, no causative microorganisms for HPV-negative OSCC have been identified. Whether causative or not, continuous bathing of a developing carcinoma in microbial products is a potentially important factor, in part because they can trigger and/or support chronic inflammation. The role of colonizing microbes in OSCC pathogenesis has not been defined.

The recognition of microbial products, irrespective of pathogenicity, is generally a function of pattern recognition receptors (PRR). A subset of PRR known as Toll-like receptors (TLR) is used by antigen presenting and other immune system cells. In immune system cells, activated TLR lead to inflammation via the canonical NF-kB pathway and other signals [11]. A key pathway induced by nearly all TLR (except TLR3) in various immune cells is MyD88-dependent and involves the phosphorylation of MAPK3 ERK1 and MAPK1 ERK2, which then translocate to the nucleus and activate transcription factors [11, 12].

In the context of the G^pos^ and G^neg^ bacteria colonizing OSCC [4–9], cell surface TLR2 and TLR4 are of particular interest, because LPS from most G^neg^ bacteria activates TLR4, while various products (lipopeptides and peptidoglycans) from G^pos^ bacteria primarily activate TLR2 [11, 13]. A unique feature of TLR2 is the very broad range of ligands, because it dimerizes with two other TLR, TLR1 (TLR2/1) or TLR6 (TLR2/6), and can partner with other co-receptors [11, 13]. We and others have observed that while normal oral squamous epithelium is relatively unresponsive to TLR stimuli, TLR2 and TLR4 ligands often induce SCC cells to make pro-tumor factors, such as IL-6, IL-8, VEGF, CCL2 [14–16]. TLR4 activation in head and neck SCC was shown to promote cell survival and growth, and even offer protection from cisplatin-induced apoptosis [16]. A role for cell-intrinsic TLR2 and MyD88 in intestinal and breast epithelial (non-squamous) cells and oncogenesis has been described [17, 18]. Remarkably, epithelial cell TLR2, independent of its expression in hematopoietic tumor-infiltrating cells, was shown to directly promote gastric adenocarcinoma growth in mice [19], while expression and signaling of TLR2 in epithelial cells of the small intestine was dependent on microbial colonization of the gut in mice [18]. In addition, *high* TLR2 expression common particularly in human intestinal-type gastric adenocarcinoma, was shown to be associated with a certain TLR2-regulated gene profile and poor patient outcomes [20]. Another recent study suggested that TLR2 may also be important for OSCC cells, because blocking TLR2 inhibited tumor growth in a xenograft immunodeficient mouse model [21]. Yet, the function of TLR in OSCC is largely unknown.

Unchecked TLR activation can lead to severe inflammation with tissue damage. The damage is controlled in part via inhibitory adenosine receptors (AR), which are members of the G-protein-coupled receptor family. A major source of adenosine at sites of inflammation and in the cancer microenvironment, including head and neck SCC [22], is extracellular ATP, which is released from stressed or dying cells and de-phosphorylated by cell surface enzymes [23–25]. Adenosine acts via differentially expressed AR A1, A2a, A2b and A3 [24, 26]. In contrast to A1 and A3, A2a (and to some extent, the low-affinity AR A2b) inhibits destructive inflammation by inducing cyclic AMP, while promoting regulatory T cells and wound healing [24, 26–28]. In immune system cells, TLR activation causes a decrease in A1 and A3, while A2a expression is increased and it acts as a key inhibitor of immune system cell inflammatory responses [23]. Similar to the MyD88-dependent pathway of TLR activation, A2a signals induce MAPK3/1 ERK1/2 phosphorylation in immune system cells [23], which then results in suppression of proinflammatory cytokines via phosphorylation of c-FOS [29].

To address the gap in the understanding how OSCC cell TLR and AR affect malignant squamous cells, we characterized the expression and function of TLR2, TLR4 and AR in OSCC cells. We show that *in vivo*, keratinocytes in epithelial dysplasia and in OSCC expressed both TLR2 and A2a. *In vitro*, OSCC cells expressed TLR1, 2, 4 and 6 and responded to their ligands. OSCC cells also expressed inhibitory AR A2a and A2b, but not stimulatory A1 or A3. TLR2-high cells upregulated A2a, but not A2b in response to TLR2 ligands, while TLR4 failed to affect AR expression in nearly all instances. TLR2 and AR activities induced phosphorylation of ERK1/2 in OSCC cells. TLR2 stimuli in TLR2-high OSCC lead to c-FOS accumulation followed by ERK-dependent proliferation and reduced caspase-3 activity. Similar lack of caspase-3 activity and ERK1/2-dependent proliferation of TLR2-high OSCC was caused by combined TLR2 and AR stimuli. The implications of these results are that persistence of microorganisms and adenosine characteristic of the OSCC tumor microenvironment may directly promote the activation of oncogenic pathways in OSCC cells.

## RESULTS

### TLR2 and TLR4 stimuli activate the NF-kB pathway in OSCC cells

We reported previously that OSCC cells express TLR4 and CD14 and can produce cytokines and/or chemokines in response to TLR4 stimuli, but primary or telomerase-immortalized oral keratinocytes are relatively unresponsive [14]. We also found that OSCC cell lines were more likely to secrete NF-kB-driven factors in response to TLR2/1 than TLR4 stimuli (not shown). RNA analysis confirmed that in OSCC cells, TLR2/1 stimuli activated the MyD88-IRAK2-NF-kB pathway and induced NF-kB-dependent cytokines and chemokines, without a similar effect on oral keratinocytes (Table 1). Interestingly, baseline TLR2 mRNA expression in some OSCC cells was higher than TLR4, TLR1, or TLR6 mRNA, similar to positive control THP1 cells. OSCC cells UMSCC19 and oral keratinocytes expressed more TLR4 mRNA than TLR2, and UMSCC19 also expressed the highest TLR6 levels of all TLR analyzed (Supplementary Table 1).

**Table 1:** Distinct patterns of mRNA expression in human oral keratinocytes (KER), OSCC and DC stimulated via TLR4 (LPS), TLR2/1 (P3CSK4), or TLR4+2/1

Genes	Cal27			hTERT HAK Clone 41			DC
	TLR stimuli			TLR stimuli			TLR stimuli
	TLR2/1	TLR4	TLR2/1+TLR4	TLR2/1	TLR4	TLR2/1+TLR4	TLR2/1+TLR4
MYD88	**2.1**	**4.1**	**2.3**	−1.9	−1.1	1.2	**6.1**
NFKB1	1.9	**2.3**	**2.4**	1.3	1.1	−4.5	**157.6**
NFKB2	−1.5	−1.1	−1.0	−2.4	−1.2	−5.2	**16.4**
NFKBIA	**3.6**	**5.2**	**4.8**	1.1	1.1	−6.8	**65.8**
NFKBIL1	−2.0	−1.1	−1.0	−1.2	1.3	**3.2**	**5.0**
NFRKB	−1.4	−1.3	−1.2	−1.4	1.2	1.4	**2.9**
TICAM2	−1.3	1.0	−1.2	−1.4	−1.3	1.1	**21.9**
TIRAP	−1.5	−1.3	−1.2	−1.2	1.3	1.6	**3.3**
TNF	**4.9**	**10.1**	**6.9**	1.0	−1.2	1.3	**1341.8**
CCL2	**9.7**	**18.5**	**14.9**	−1.1	1.1	−1.1	**60.5**
IL8	**9.1**	**14.2**	**10.8**	−1.2	1.2	1.3	**1541.4**
IL6	**2.4**	**3.6**	**2.4**	1.2	1.4	1.0	**492580.5**
IL1A	**2.5**	**4.4**	**2.7**	−1.1	1.1	−1.0	**5752.6**
IL1B	1.9	3.3	1.8	−1.1	1.1	1.0	**39786.7**
IRAK1	−2.0	−1.5	−1.4	−1.4	1.3	1.8	**5.6**
IRAK2	**3.1**	**4.8**	**4.1**	−1.2	1.1	1.4	**340.1**

Telomerase-immortalized oral keratinocytes hTERT HAK Clone 41 and SCC cells Cal27 were incubated for 4 hrs with or without highly pure TLR4-specific *E. coli* LPS (300 U/ml) and/or TLR2-specific Pam3CysSerLys4 (P3CSK4, 300 ng/ml). Similarly, DC were stimulated with TLR4+2/1 agonists (positive controls). Total RNA was purified using RNAqueous-4PCR kit (Applied Biosystems) and evaluated for quantity and purity, followed by cDNA synthesis from 0.5 μg of each RNA sample using the RT^2^ First Strand Kit (SABiosciences). Real-Time PCR using a three-step cycling protocol was performed with the RT^2^ Profiler PCR Array Human Toll-Like Receptor Signaling Pathway system (SABiosciences) and the MJ Research Opticon 2 thermocycler. *The numbers represent fold change in expression relative to unstimulated cells*. Selected genes show effects on TLR signaling pathways (MyD88, IRAK2, TICAM, NFKB) and expression of inflammatory products and chemokines regulated by NFKB (CCL2, IL-1b, IL-8).

These data indicate that TLR2 and TLR4 stimuli activate the NF-kB pathway in malignant squamous cells, similar to myeloid dendritic cells, and raise a possibility that TLR activity in OSCC cells could affect the expression of AR.

### Human OSCC cells do not express AR A1 or A3, and TLR2 activation stimulates A2a expression

Further mRNA analysis showed that in contrast to THP1 cells, keratinocytes and OSCC cells had no detectable A1 or A3 mRNA (Supplementary Table 1). However, all cells expressed A2a and A2b, while A2b levels were higher than A2a at baseline. Control THP1 cells, as predicted, upregulated A2a and A2b and downregulated A1 and A3 when stimulated via TLR2 or TLR4 (Table 2). TLR2/1 agonist Pam3CSK4 consistently stimulated A2a expression in five out of six OSCC cell lines with high TLR2 levels, but not in TLR2-low immortalized oral keratinocytes or in UMSCC19 carcinoma cells. TLR4-specific LPS stimulated A2a expression only in one out of six OSCC lines. A2b expression in OSCC cells often decreased upon TLR2/1 stimulation, in contrast to that in control THP1 cells.

Table 2TLR2/1 stimuli (A) induce upregulation of A2a in most OSCC cell lines, while TLR4 stimulus (B) fails to induce A2a in most OSCC cells

**Table:** 

(A) TLR2/1 stimulation (P3CSK4)				
Cell lines	A1	A2a	A2b	A3
**THP1**	0.6 ± 0.8	151 ± 8.4^***^	1.7 ± 0.04^***^	0.8 ± 0.05^*^
**keratinocytes**	none	1.2 ± 0.2	0.9 ± 0.1	none
**PCI13**	none	11 ± 0.8^***^	0.6 ± 0.04^***^	none
**Cal27**	none	6.4 ± 0.7^***^	0.75 ± 0.03^***^	none
**FaDu**	none	1.9 ± 0.1^***^	0.9 ± 0.01	none
**SCC4**	none	2.5 ± 0.2^***^	0.98 ± 0.01	none
**UMSCC1**	none	2.2 ± 0.2^***^	0.9 ± 0.04^***^	none
**UMSCC19**	none	1.3 ± 0.2	1 ± 0.02	none

Fold change relative to unstimulated cells, ± SD.

Bold = significant; ^*^p < .05; ^**^p <. 01; ^***^p < .001.

**Table:** 

(B) TLR4 stimulation (*E. coli* LPS)				
Cell lines	A1	A2a	A2b	A3
**THP1**	0.44 ± 0.25^*^	169 ± 13.5^***^	1.7 ± 0.09^*^	0.8 ± 0.03^*^
**keratinocytes**	none	1.0 ± 0.27	0.98 ± 0.07	none
**PCI13**	none	1.04 ± 0.18	1.04 ± 0.09	none
**Cal27**	none	1.32 ± 0.07	0.94 ± 0.07	none
**FaDu**	none	1.25 ± 0.09^***^	1.0 ± 0.02	none
**SCC4**	none	0.96 ± 0.14	0.93 ± 0.00^**^	none
**UMSCC1**	none	0.97 ± 0.09	0.95 ± 0.02	none
**UMSCC19**	none	1.24 ± 0.21	0.94 ± 0.01^*^	none

Fold change relative to unstimulated cells, ± SD.

Bold = significant; ^*^p < .05; ^**^p < .01; ^***^p < .001.

Monocytoid THP1 cells (positive control), keratinocytes hTERT HAK Clone 41, and six OSCC cell lines were stimulated for four hours with P3CSK4 (TLR2/1) or *E. coli* LPS (TLR4), and AR mRNA expression was measured by qRT-PCR, as described in Materials and Methods. Fold changes relative to unstimulated cells ± standard deviations (SD) are shown. SD include: two separate stimulations and two PCR runs for each stimulation. Data from 2–5 experiments per cell line were analyzed using ANOVA, including Tukey-Kramer test for multiple comparisons.

Together, these data indicate that in OSCC cells, only inhibitory AR A2a and A2b have the potential to react to adenosine; moreover, TLR2 is more likely than TLR4 to modulate inhibitory AR expression.

### OSCC and dysplastic epithelial cells co-express TLR2 and A2a *in vivo*

**Figure 1:**
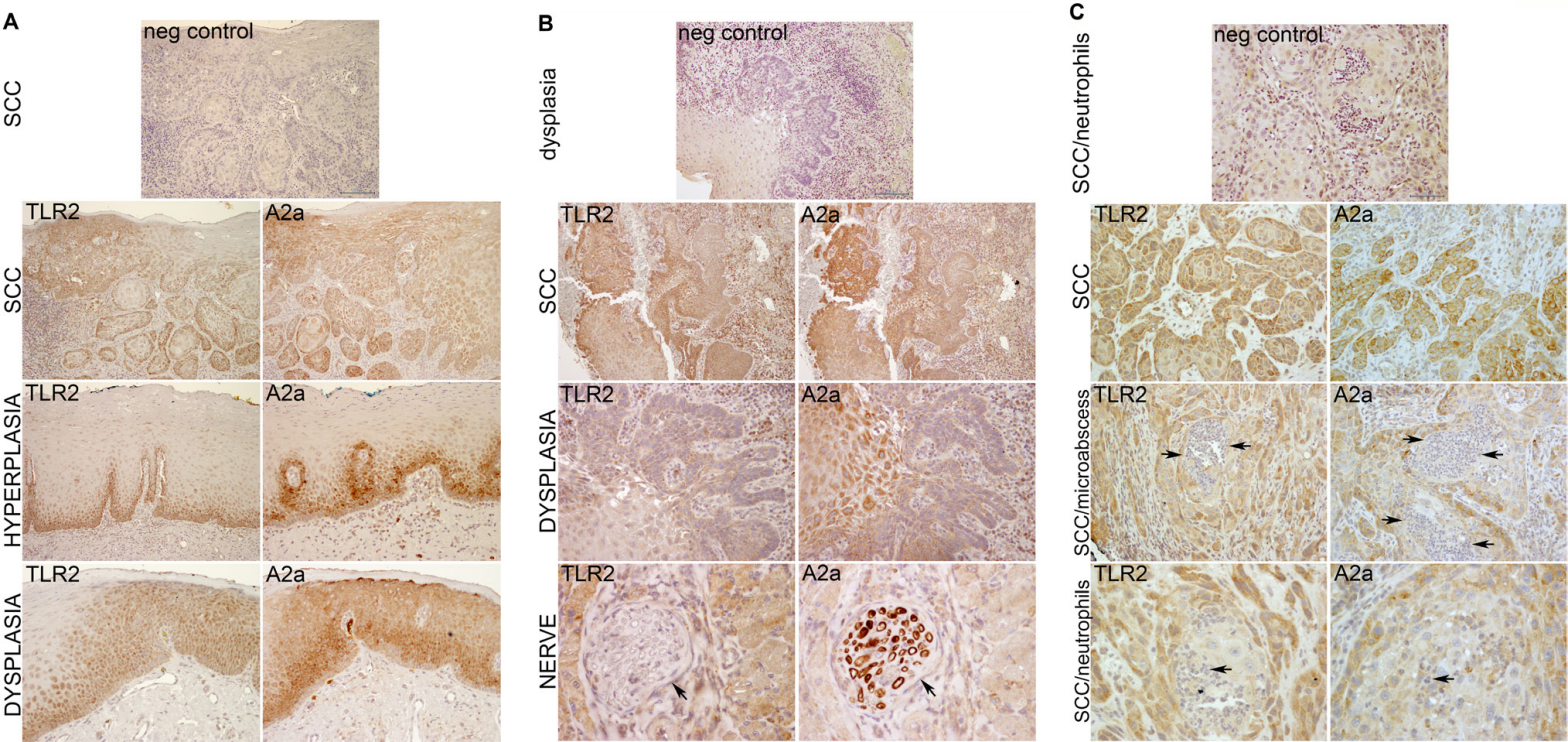
TLR2 and A2a are expressed together in oral ED and SCC cells Examples of SCC, two from the tongue (**A**, **B**) and one from the gingiva (**C**). Serial (back-to-back) sections of archival paraffin-embedded human samples were processed and stained by IHC as described in Materials and Methods. The TLR2-positive and A2a-positive cells are stained brown and nuclei are blue. Note similar expression of TLR2 and A2a throughout the dysplastic epithelium and SCC. An internal control, nerve fibers, are A2a-positive and TLR2-negative (B, Lower row, arrows). For negative controls (top panels), primary TLR2- and A2a-specific antibodies were replaced with species-, isotype- and concentration-matched nonspecific antibodies.

In order to verify the relevance of TLR2 and A2a to SCC *in vivo*, we evaluated the expression of these receptors in 17 archival specimens of human epithelial dysplasia/OSCC. Figure 1 shows representative images of three samples from the tongue and the gingiva. TLR2 and A2a were expressed in all samples, and often co-expressed in the same squamous cells. Normal or hyperplastic epithelium showed TLR2 and A2a staining in the basal and parabasal cells (Figure 1A, panel “Hyperplasia”), while TLR2- and A2a-positive squamous cells in epithelial dysplasia (ED) and in OSCC were present throughout the lesions. There was relatively little staining of neutrophils present within the gingival sample (Figure 1C), and a peripheral nerve was clearly A2a-positive (Figure 1B), which provided a strong internal positive controls for A2a. The levels of expression cannot be quantified in IHC-stained sections, but the variation in staining intensity within each sample is consistent with variation in expression. Frequently, the less differentiated, smaller cells stained more intensely than large, better differentiated cells, which may be due either to a decrease in receptor expression with cell differentiation, or to be a function of receptor dilution because of larger cell volume.

Overall, these results indicate that the expression and functions of TLR2 and A2a in squamous epithelium may be linked and are likely to be relevant throughout oral squamous carcinogenesis.

### TLR2 stimuli activate MAPK ERK1/2 and lead to early c-FOS accumulation in TLR2-high OSCC

TLR2 and A2a each activate ERK1/2 in immune system cells [11, 12, 23], and TLR2 was shown to trigger ERK1/2 phosphorylation in murine gut (non-squamous) epithelium [18]. In squamous epithelial cells these MAPKs are is activated via the epidermal growth factor receptor (EGFR), but the roles of TLR2 and AR signaling have not been determined. For further analysis, we selected two TLR2-high/responsive OSCC lines PCI13 and Cal27 and the TLR2-low/unresponsive UMSCC19 cells and keratinocytes.

**Figure 2:**
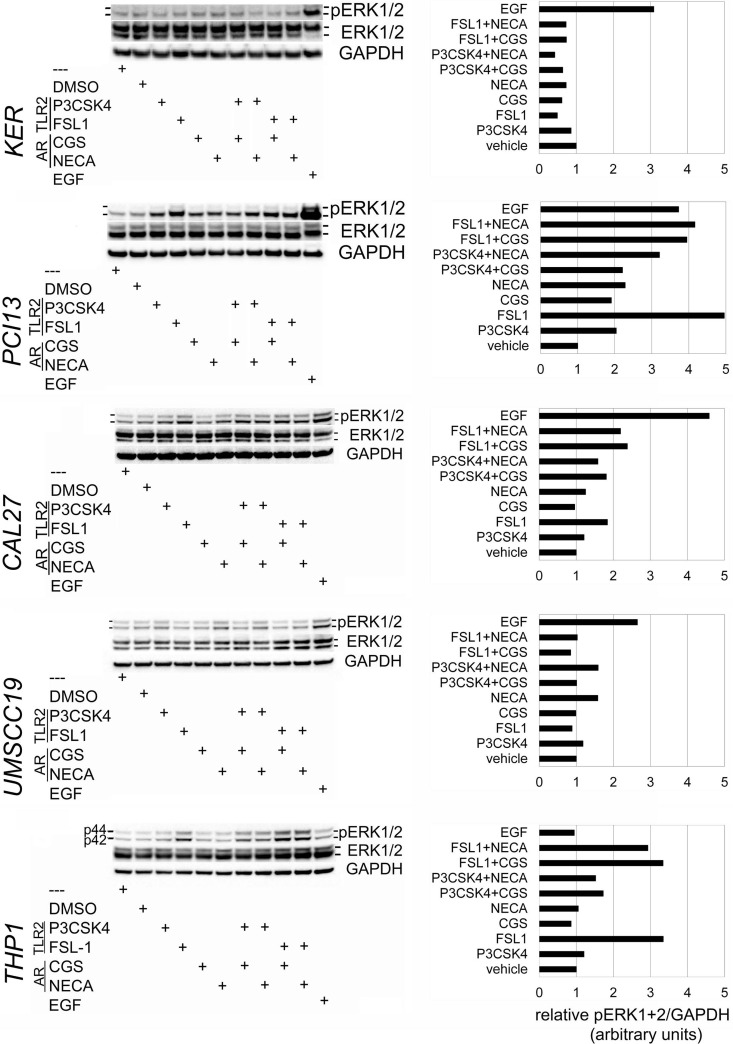
TLR2/1, TLR2/6 and A2a stimulate MAPK ERK1/2 phosphorylation in OSCC cells OSCC cells Cal27, PCI13 and UMSCC19, keratinocytes hTERT HAK Clone 41, and monocytoid THP1 cells were incubated × 15 min with EGF, TLR2/1 ligand Pam3CSK4, TLR2/6 ligand FSL-1, A2a-selective agonist CGS21689, non-selective AR agonist NECA, as detailed in Materials and Methods. The A2a-selective antagonist ZM 241385 was added in some experiments four hours prior to other stimuli. Protein was analyzed as described in Materials and Methods. Briefly, samples were separated by SDS-PAGE electrophoresis, transferred to nitrocellulose membrane, and probed for pERK1/2, total ERK1/2, and GAPDH. Charts are representative of the blots shown. Data represent up to 4 independent experiments for each cell line.

Representative results are shown in Figure 2. In positive control THP1 cells, ERK1/2 activation was induced through TLR2/1 and more so through TLR2/6, with or without AR agonists. As expected, EGF activated ERK1/2 in all epithelial cells, but not in THP1 cells. Consistent with our hypothesis, TLR2-high OSCC cells PCI13 and Cal27 showed ERK1/2 phosphorylation (mostly, ERK2) when stimulated through TLR2/1 or TLR2/6. Similarly, non-selective AR agonist NECA and A2a-selective CGS induced pERK1/2 to varying degrees in PCI13 and Cal27 cells. Combined TLR2 and AR stimulation usually resulted in more ERK phosphorylation than AR stimuli alone. In contrast, TLR2 agonists induced little if any pERK1/2 in TLR2-low keratinocytes and UMSCC19. Some AR-induced ERK1/2 activity was detectable in UMSCC19 cells. The A2a-selective antagonist ZM 241385 abrogated ERK1/2 activation induced by CGS or NECA (not shown), suggesting that A2a was probably the primary signaling AR.

**Figure 3:**
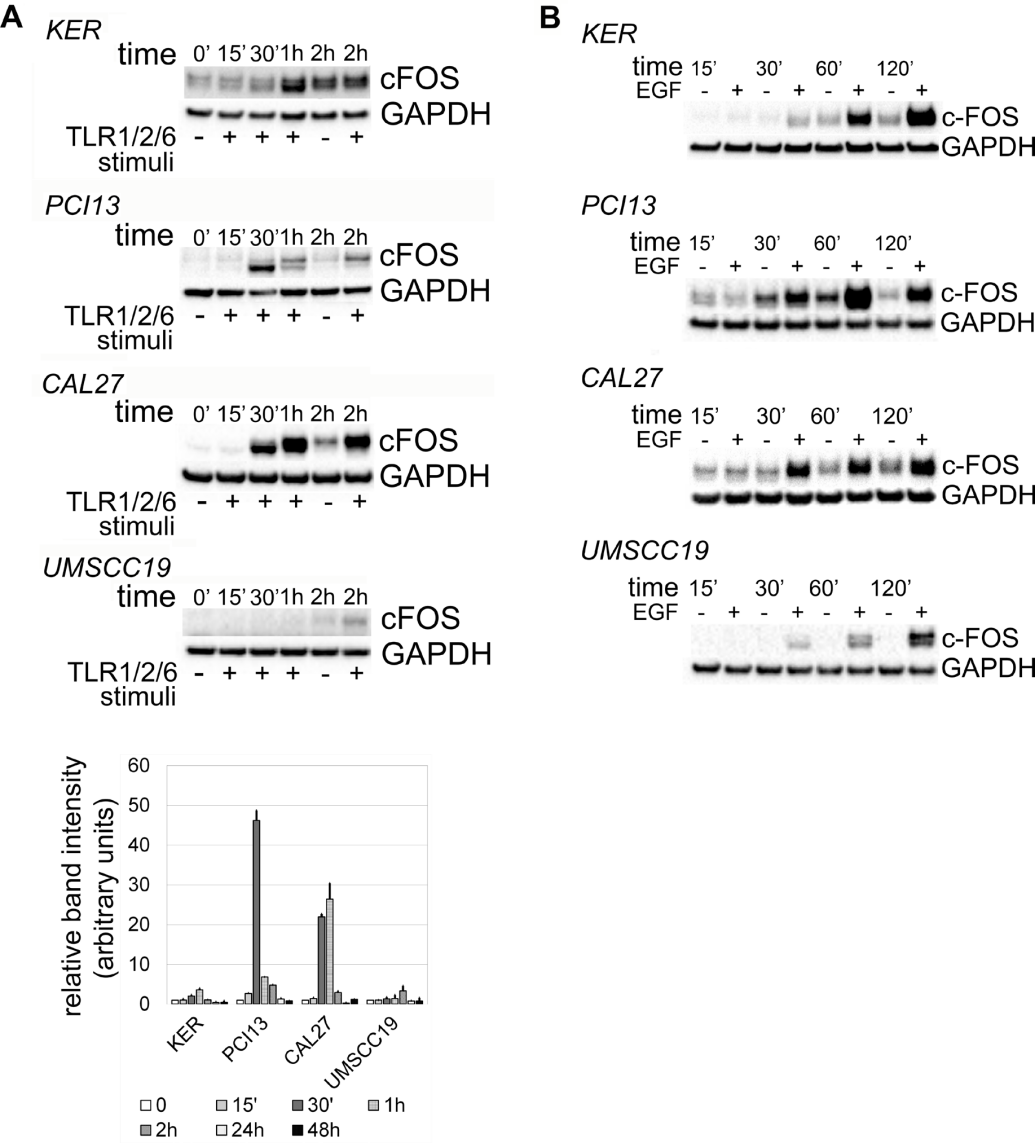
Kinetics of c-FOS accumulation in response to TLR2 stimuli (**A**) and EGF (**B**). OSCC cells Cal27, PCI13 and UMSCC19 and keratinocytes hTERT HAK Clone 41 were incubated with Pam3CSK4 and FSL-1 or with EGF for up to 48 hrs. Protein was collected at indicated time points and analyzed as described in Materials and Methods. Briefly, samples were separated by SDS-PAGE electrophoresis, transferred to nitrocellulose membrane, and probed for c-FOS and GAPDH. Mean relative densitometry values from two independent experiments, normalized to controls, are represented in the lower panel. Error bars = standard error.

The effects of activated ERK1/2 vary from induction of apoptosis or senescence to proliferation [11, 12, 23, 30–32]. The rapid accumulation of c-FOS minutes following phosphorylation of ERK1/2 reportedly supports cell proliferation [32, 33]. Interestingly, c-FOS was detected within 30 minutes of TLR2 stimuli in TLR2-high OSCC, with kinetics similar to EGFR-induced c-FOS accumulation (Figure 3). TLR2-low keratinocytes and UMSCC19 cells showed weaker and slower c-FOS kinetics in response to TLR2 signals (1 and 2 hrs, respectively). No c-FOS was detected in any OSCC cells at 24 or 48 hrs with either TLR2 or EGFR activity (not shown). Time points between two and 24 hours were not evaluated. These results suggest that in TLR2-high OSCC cells, TLR2-induced ERK1/2 activity could potentially lead to cell proliferation, which was tested as described below.

### OSCC cells proliferate in response to TLR2 ± AR stimuli, which depends upon ERK1/2 activation

**Figure 4:**
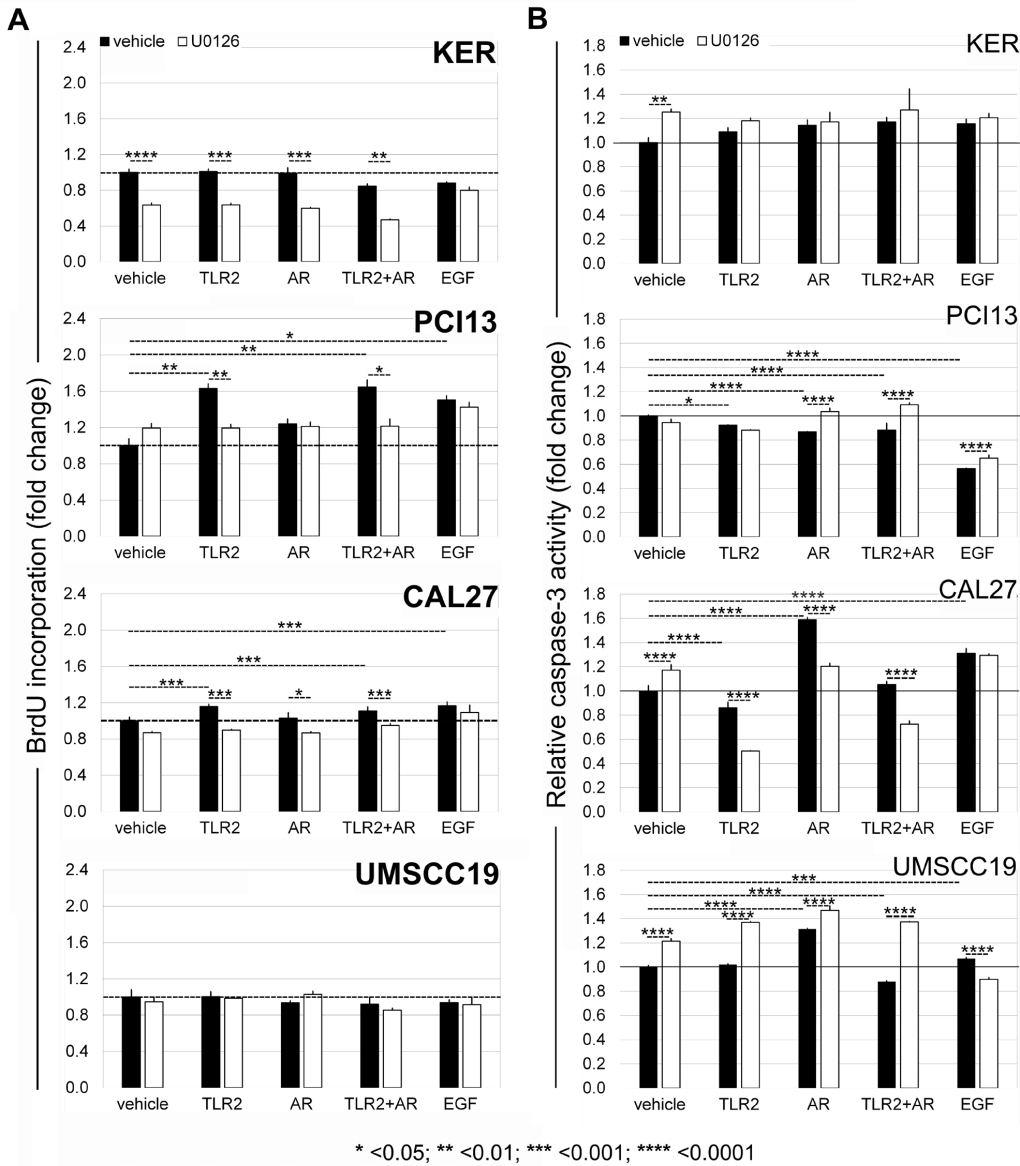
TLR2-high OSCC cells proliferate in response to TLR2 stimuli in an ERK1/2-dependent manner (**A**) without activating caspase-3 (**B**). Functional experiments were performed as described in Materials and Methods. Briefly, after titrating ERK inhibitor U0126 (Supplementary Figure 1), cells were incubated with and without TLR2/1+TLR2/6 stimuli (Pam3CysSerLys 3 and FSL-1), AR ligand NECA, or both, in the presence of absence of 1 μM U0126. (A) BrdU incorporation measured at 24 hrs as described in Materials and Methods. Values represent mean relative values normalized to unstimulated cells, two independent experiments, each in quadruplicate the four cell lines, and standard deviations. (B) Caspase-3 activity was measured as described in Materials and Methods. Charts show caspase activity normalized to that in unstimulated cells from two experiments, in triplicate or quadruplicate for each cell line. (A and B): Error bars = Standard deviations. Statistical significance of differences between groups was assessed by one-way ANOVA, including Tukey-Kramer post-tests for multiple comparisons.

The effects of TLR2 stimuli on cell proliferation were quantified by measuring the expression of Ki-67 (Table 3), a specific and sensitive marker of cycling cells [34], and followed-up with the BrdU assay (Figure 4A). EGF stimulated the expression of Ki-67 mRNA in keratinocytes, PCI13 and Cal27 cells. By 24 hrs, all four cell lines upregulated Ki-67 in response to TLR2 stimuli, more pronounced in TLR2-high PCI13 and Cal27, suggesting that entry into the cell cycle may be triggered even in OSCC cells with relatively low TLR2 expression. To test A2a and A2b AR activity, both of which are inhibitory, but the relative levels of which can change with TLR stimulation, we employed AR-nonselective agonist NECA. NECA alone or combined with TLR2 stimulation induced Ki-67 expression without a significant impact on keratinocytes or UMSCC19 cells (Table 3). Moreover, TLR2-high, but not TLR2-low cells, increased DNA synthesis in response to TLR2 activity, which returned to baseline in the presence of ERK inhibitor (Figure 4A and Supplementary Figure 1). Interestingly, proliferation responses as measured by Ki-67 and BrdU were very similar, whether TLR2 stimuli were provided alone or in combination with AR agonists. While NECA alone stimulated Ki67 expression, it did not significantly impact DNA synthesis at 24 hrs. Together, these results support the conclusion that TLR2 with or without AR engagement can stimulate OSCC cell proliferation in TLR2-high cells, at least in part, via the MAPK ERK1/2 pathway, and that TLR2 signaling dominates responses to combined TLR2-AR stimuli.

**Table 3:** TLR2 and/or AR stimuli induce Ki-67 mRNA expression in OSCC cells by 24 hrs

*stimuli*	KER	PCI13	CAL27	UMSCC19
**none**	1.00 ± 0.02	1.00 ± 0.03	1.00 ± 0.05	1.00 ± 0.02
**TLR2/1+2/6**	1.15 ± 0.03^*^	3.02 ± 0.63^***^	1.52 ± 0.04^****^	1.11 ± 0.02^**^
**AR**	1.08 ± 0.07	2.03 ± 0.35^*^	1.45 ± 0.003^****^	1.03 ± 0.04
**TLR2/1+2/6+AR**	0.94 ± 0.08	1.49 ± 0.27^*^	2.11 ± 0.05^****^	1.01 ± 0.04
**EGF**	1.52 ± 0.04^****^	3.14 ± 0.78^***^	1.42 ± 0.05^****^	0.70 ± 0.02^**^

Fold change relative to unstimulated cells, ± SD.

Bold = significant; ^*^p < .05; ^**^p < .01; ^***^p < .001; ^****^p < .0001.

OSCC cells Cal27, PCI13 and UMSCC19, and keratinocytes hTERT HAK Clone 41 were incubated for 2, 4, and 24 hrs with EGF, or with one or more of the TLR2/1, 2/6 ligands and AR agonist NECA, as indicated in Materials and Methods. Cellular mRNA was evaluated for Ki-67 and GAPDH expression by qRT-PCR, in triplicate. Fold changes relative to unstimulated cells ± SD are shown. Data were analyzed using one way ANOVA, including Tukey-Kramer test for multiple comparisons.

### TLR2 signaling does not stimulate caspase-3 activity in TLR2-high OSCC cells

We also addressed the possibility that ERK activation may induce apoptosis of SCC cells. Procaspase-3 is cleaved to produce active caspase-3, which is critical for the apoptotic cascade [35]. Notably, EGFR stimulation significantly suppressed active caspase-3 in PCI13 cells, but in Cal27 and UMSCC19 cells these levels increased. The most important observation was that in TLR2-high cells, TLR2 with or without AR ligation either decreased caspase-3 activity, or did not increase it (Figure 4B). The role of ERK1/2 in these outcomes was not clear, because the effect of ERK inhibitor varied. Overall, the data support the interpretation that in TLR2-high OSCC cells, TLR2 with or without AR stimuli did not induce apoptotic cell death, and may have been protective. Together with Ki67 and BrdU data, these results indicate that TLR2-high OSCC cells can receive growth-promoting signals through the TLR2-induced ERK1/2 pathway while maintaining viability. A summary of the TLR2-mediated effects in keratinocytes and OSCC cells is provided in Table 4.

**Table 4:** Summary of TLR2 expression levels and TLR2-mediated effects on squamous cells tested in this study

	KER	PCI13	CAL27	UMSCC19	FaDu	SCC4	UMSCC1
**TLR2 baseline level**	low	high	high	low	high	high	high
**Upregulation of A2a**	no	yes	yes	no	yes	yes	yes
**Downregulation of A2b**	no	yes	yes	no	no	no	yes
**Expression of A1 and A3**	none	none	none	none	none	none	none
**ERK1/2 activation**	no	yes	yes	no	ND	ND	ND
**cFOS at 30’**	no	yes	yes	no	ND	ND	ND
**Increase in Ki67**	yes (min)^**^	yes	yes	yes (min)^**^	ND	ND	ND
**ERK1/2-dependent increase in BrdU incorporation**	no	yes	yes	no	ND	ND	ND
**caspase-3 activity**	increase?^*^	decrease	decrease	no change	ND	ND	ND

^*^ Not statistically significant; ^**^min = minimal. ND = not done.

## DISCUSSION

Our study adds important insights to the increasing body of literature that identifies a unique role for TLR2 in inflammation and cancer. These observations are important for the understanding of OSCC biology, because these cancers are raised in a microenvironment rich in products of microorganisms and inflammation. We hypothesized that the activation of TLR2 in OSCC cells is similar to that in immune system cells, including the growth and survival-promoting ERK1/2 activity and the interactions with AR. A key finding is that TLR2, with or without inhibitory AR activation, can directly stimulate proliferation of TLR2-high malignant oral squamous cells via the pro-oncogenic MAPK ERK1/2 pathway, as well as promote survival of these cells, thus potentially allowing some level of tumor cell autonomy from inflammatory cells in the context of microbial colonization and inflammation.

The functional TLR2 expression in head and neck SCC has been shown in other studies. An important observation was made by Farnebo et al, who reported that blocking TLR2 inhibited SCC growth in a xenograft immunodeficient mouse model [21], suggesting that TLR2 may stimulate squamous carcinoma growth. Our results are in agreement and indicate that SCC cell growth is inducible via the ERK1/2 pathway in cells with high TLR2 expression, i.e. in cells that have more TLR2 than TLR4 (Supplementary Table 1). Both TLR2/1 and TLR2/6 ligands activated ERK1/2, though higher TLR6 expression than TLR1 may have contributed to the stronger effect of the TLR2/6 ligand FSL-1 than the TLR2/1 ligand Pam3CysSerLys2. In this regard it is of interest that, TLR2/6 is also activated by a host matrix protein versican [36], the expression of which predicts progression and poor prognosis in oral cancer [37, 38]. Which microorganisms contribute to OSCC development and growth is unresolved, although recent studies suggest a reduction in diversity of commensal species with an increase in G^pos^ *Streptococcus Mitis*, G^neg^ *Prevotella melaninogenica,* and G^neg^ *Capnocytophaga* at OSCC sites [8, 9]. Other studies have suggested a possible role for opportunistic periodontal pathogens *Porphyromonas gingivalis* and *Fusobacterium nucleatum* [39, 40], which can activate multiple TLR, including TLR2.

The cancer microenvironment contains both TLR ligands and adenosine. Moreover, TLR2 and AR functions in immune system cells are linked, including their ability to activate MAPK ERK1/2. Immune cell TLR2 stimulates the expression of inhibitory A2a and A2b, such that adenosine binding suppresses excessive toxic type I inflammation associated with TNF-alpha production, while promoting wound-healing mechanisms [23, 41]. Our study shows that TLR2 effects on AR expression in OSCC cells only partially replicate those in immune cells. Remarkably, keratinocytes and OSCC cells had only inhibitory A2a and A2b receptors, but no detectable stimulatory A1 or A3, and TLR2 stimulated the expression of A2a, while A2b expression sometimes decreased.

The expression of inhibitory AR in keratinocytes may be important for normal surface barrier function, because activated keratinocytes can produce TNF-alpha that may compromise the barrier [9], and it is interesting that OSCC cells retain this A1- and A3-negative phenotype. Similar to immune cells, both nonselective AR agonist and A2a-specific agonist induced ERK phosphorylation in OSCC cells. Additional experiments with A2a antagonist ZM 241385 suppressed ERK activation and suggested that A2a in the absence of TLR stimulation was probably the dominant of the two AR, at least in the normoxic conditions we used. Given that both OSCC AR are inhibitory, and that their expression is subject to change with TLR stimulation, further dissecting specific A2b contributions in OSCC cells may be of limited value. However, because hypoxic conditions in established cancer microenvironments can result in high adenosine concentrations, A2b signaling could be important [23, 25].

AR functions in malignant epithelial cells are still sketchy. In contrast to our results with squamous cells, non-squamous (adenocarcinoma) cell lines of the stomach [42] and colon [43] were reported to express A1 and A3 and underwent apoptosis induced via A1, while agonist-mediated A3 activation was shown to cause cell cycle arrest and apoptosis in adenocarcinoma cell lines from the lung [44] and liver [45]. Apoptosis of colon adenocarcinoma cells Caco-2 [41] and hepatocellular carcinoma cells HepG2 [42] was reportedly induced via A2a AR, and activated A2b was found to induce apoptosis in a p73-dependent manor in transfected cancer cells [43]. These studies did not address combined TLR/AR signaling.

A major source of adenosine at sites of inflammation and in the cancer microenvironment, including head and neck SCC [22], is extracellular ATP released from stressed or dying cells [23–25]. Whether microorganisms that colonize OSCC also release ATP in the tumors is not known. ATP is sequentially dephosphorylated to AMP by cell-surface enzyme CD39 (ecto-nucleoside triphosphate diphosphohydrolase 1, E-NTPDase1), followed by AMP de-phosphorylation to adenosine by CD73 (ecto-5’-nucleotidase, Ecto5’NTase) [23]. Our previous studies showed that OSCC cells selectively suppressed the production of TNF-alpha by lipopolysaccharide-stimulated monocytes *in vitro* [14], consistent with adenosine-mediated activity. Moreover, similar to monocytes, OSCC cells express both CD39 and CD73 (data not shown). Although adenosine is relatively unstable because of the enzyme adenosine deaminase, it turns out that the degradation product inosine, a much more stable molecule, is also an A2a agonist [46, 47].

Analysis of the downstream effects of TLR2-induced ERK1/2 activation is important because of the wide range of potential outcomes from apoptosis to survival, from differentiation and senescence to proliferation, and other effects [31–33, 48]. ERK1/2-mediated stabilization of the AP-1 component c-FOS is associated with cell survival and proliferation [31, 32, 49, 50]. We detected c-FOS in TLR2-high OSCC cells minutes after TLR2 stimulation, and its changing electrophoretic mobility was consistent with c-FOS phosphorylation [33, 51–53]. We showed that c-FOS was stabilized for no less than 90 minutes in TLR2-responsive OSCC, which was comparable to the EGF-induced c-FOS activity. Therefore, cell proliferation was the predicted outcome, which was confirmed using Ki-67 analysis [34] and the BrdU assay. We saw increases both in Ki-67 mRNA and BrdU incorporation in response to TLR2 stimuli with or without AR agonists, similar to EGF effects, strongly correlating with the pERK1/2 and c-FOS data. On the other hand, AR stimulation alone induced Ki-67, without significant BrdU incorporation at 24 hrs, which could be due to differences in assay targets: Ki-67 is more sensitive, because it is expressed throughout the entire cell cycle, while BrdU is only incorporated during the S phase, so more cycling cells are detectable when Ki-67 is measured vs. BrdU at 24 hrs [54].

In distinction from TLR2-mediated effects, independent AR stimuli induced pERK and in some cases caspase-3 activity increased. It is interesting that besides inducing apoptosis, caspase-3 activity may trigger a so-called bystander PGE_2_-mediated proliferation of neighboring cells [55, 56], which would be important to investigate in OSCC. Yet, the outcomes of combined TLR2+AR signals were clearly dominated by TLR2 activity and resulted in similar cell proliferation and lack of caspase-3 activation to those in response to TLR2 only signals. Based upon the similar expression of TLR2 and AR A2a in many malignant cells of OSCC samples, co-expression of the two receptors is probably common, though may be limited to a subset of tumor cells. One possibility is that the levels of TLR and AR expression and the outcomes of their signaling could vary with the level of cell differentiation. Overall, our data suggest that TLR2-high cells co-expressing inhibitory AR would likely be stimulated to proliferate and resist apoptosis.

An important pathway activated through TLR2 in many cells, including OSCC, is NF-kB. Moreover, there are indirect effects of NF-kB activity, whereby induced soluble factors may act on their own receptors in an autocrine manner, and time points beyond 24 hrs could help reveal the indirect effects. For example, some OSCC produce IL-6 in response to TLR stimuli, which activates IL-6 receptors in OSCC cells, leading to STAT3 activation [14] with an important cytoprotective effect. Besides other pro-cancer properties, activated STAT3 was shown to stimulate the expression of TLR2 in gastric carcinoma cells [19]. If a similar mechanism is true in OSCC cells, TLR2 impact on carcinoma cells could be amplified. Further studies are needed to evaluate this possibility.

We conclude that in the tumor microenvironment, products that stimulate TLR2 and inhibitory AR may contribute to OSCC progression in part by directly activating the MAPK ERK1/2 pathway in malignant cells.

## MATERIALS AND METHODS

### Reagents and antibodies

Phosphatase inhibitor and bovine serum albumin (BSA) fraction V (protease free) were purchased from Roche Diagnostics (Indianapolis, IN). Bicinchoninic acid (BCA) assay kit, Chemiluminescent Substrate kit, fetal bovine serum (FBS), PBS, and HBSS were obtained from Thermo Scientific (Rockford, IL). Bis-Tris Plus gradient gel and i-blot membranes were purchased from Life Technologies (Grand Island, NY). Tween-20 and dimethyl sulfoxide (DMSO) were obtained from Fisher Scientific (Pittsburgh, PA). Pam3CysSerLys4 (TLR1/2-specific synthetic triacylated lipoprotein) and FSL-1 (TLR2/6-specific *Mycoplasma salivarium*-derived synthetic diacylated lipoprotein) were purchased from Invivogen (San Diego, CA, USA). Highly-pure lipopolysaccharide (LPS) of *Escherichia coli* was purchased from Sigma-Aldrich (St. Louis, MO). Adenosine receptor agonists CGS 21689 hydrochloride (A2a-selective) and NECA (non-selective), as well as adenosine receptor A2a-selective antagonist ZM 241385 were purchased from Tocris Biosciences (Bristol, UK). Cell lysis buffer and phenylmethylsulfonyl fluoride (PMSF) were purchased from Cell Signaling Technology (Boston, MA, USA). Dulbecco's Modified Eagle Medium (DMEM), nutrient mixture F-12, Keratinocyte Serum Free Medium (KSFM) with supplements and EGF were purchased from Invitrogen (Carlsbad, CA).

Primary mouse monoclonal antibodies (Ab) against phospho-p44/p42 ERK1/2 (Thr 202/Tyr 204), rabbit polyclonal Ab against total p44/p42 ERK 1/2, and rabbit monoclonal Ab against c-FOS were purchased from Cell Signaling Technology (Danvers, MA). Rabbit polyclonal Ab against adenosine receptors A2a (ADORA2A) and A3 (ADORA3) were purchased from AVIVA Systems Biology (San Diego, CA, USA). Mouse monoclonal Ab against Glyceraldehyde-3-Phosphate Dehydrogenase (GAPDH) was received from Meridian Life Science, Inc. (Memphis, TN). The goat-anti-rabbit (GAR) poly-horseradish peroxidase (HRP) secondary antibody was purchased from Thermo Scientific, and goat anti-mouse (GAM) IgG-HRP-conjugated secondary Ab was obtained from Bio-Rad (Hercules, CA, USA).

The following RT-PCR primers were purchased from Life Technologies: Hs00413978_m1 (TLR1), Hs01872448_s1 (TLR2), Hs00152939_m1 (TLR4), Hs01039989_s1 (TLR6), Hs00181231_m1 (AR A1), Hs00169123_m1 (AR A2a), Hs00386497_m1 (AR A2b), Hs00252933_m1 (AR A3), Hs9999905_m1 (GAPDH), Hs01032443_m1 (Ki-67), Hs00174103_m1 (IL-8). Additional reagents are described with the methods.

### Cells

Head and neck (oral) squamous carcinoma cell lines used were Cal-27 (tongue), FaDu (pharynx) and SCC4 (tongue) (ATCC, Rockville, MD, USA), UMSCC1 (floor of mouth) and UMSCC19 (tongue) (Dr. T. Carey, U. Michigan, Ann Arbor, MI, USA); and PCI13 (oral cavity) (a gift from Dr. T. Whiteside, U. Pittsburg, Pittsburg, PA). The carcinoma cells were cultured in DMEM/Ham's F-12 (50/50) with 10% heat-inactivated FBS. Telomerase-immortalized tonsillar keratinocytes hTERT HAK Clone 41 (a gift from Dr. A. Klingelhutz and Dr. J. Lee, U. Iowa, Iowa City, IA, USA [57]) were cultured in KSFM with 0.2 ng/ml epidermal growth factor (EGF) and 30 μg/ml bovine pituitary extract. Our previous studies showed that these telomerase-immortalized keratinocytes (previously labeled ‘tertAd7cl41’) were functionally similar to primary oral keratinocytes [14]. Monocytoid leukemia cell line THP-1 (ATCC) was cultured in DMEM with 20% heat-inactivated FBS. All cell lines were maintained at 37°C with 7% CO_2_ in a humidified incubator and tested negative for mycoplasma, using the Universal Mycoplasma Detection Kit (ATCC). Human monocyte-derived dendritic cells (DC) were produced as described previously under a protocol approved by the NYU University Committee on Activities Involving Human Subjects [58].

### Experimental set-up

For all *in vitro* experiments, carcinoma cells and keratinocytes were plated in their normal growth media at 5 × 10^5^/well in 6-well plates for attachment and equilibration ~12 hrs prior to the experiment. The cells were washed with HBSS 30 minutes before the experiment, then treated with the indicated stimuli in DMEM/F12 + 10% FBS for specified time periods (see “Results”) at 37°C with 7% CO_2_ in a humidified chamber. THP1 cells were stimulated in their own growth medium. Reagents used in experiments included one or more stimuli, as indicated: EGF (50 ng/ml; Invitrogen), TLR2/1-specific Pam3CysSerLys4 (1000 ng/ml), TLR4-specific LPS (1000 U/ml), TLR2/6-specific FSL-1 (50 ng/ml), non-selective AR agonist NECA (1 μM), A2A-specific agonist CGS 21689 (1 μM), and A2a-specific antagonist ZM 241385 (1 μM). AR reagents were in DMSO, therefore negative controls were prepared with and without DMSO. For kinetics studies, cell lysates were collected at several time points: 0’, 15’, 30’, 1 hr, 2 hrs, 4 hrs, 24 hrs and 48 hrs. Cellular products and supernatants were collected for various analyses, including mRNA (qRT-PCR or microarrays), protein expression and signaling (Western blotting), and cytokine production (ELISA).

### Quantitative real-time polymerase chain reaction (qRT-PCR)

Total RNA was purified using RNeasy Plus Mini Kit, according to manufacturer instructions (Qiagen; Valencia, CA), and stored at −80°C. Analysis by qRT- PCR for TLR1, TLR2, TLR4, TLR6, A1, A2A, A2B, A3, Ki-67 and GAPDH mRNA expression was performed using Assays-On-Demand Gene Expression Products and the StepOnePlus real time RT-PCR system (Applied Biosystems; Life Technologies) using Express One-Step Superscript qRT-PCR Universal kit (Invitrogen) and manufacturer's instructions. Briefly, cDNA was synthesized at 50°C for 15 minutes using 25 ng of RNA and subsequently amplified at 95°C for 20 seconds to activate the Platinum^®^ Taq DNA polymerase enzyme, followed by 40 cycles of 95°C for 15 seconds and 60°C for 20 seconds. The relative expression of the gene of interest was determined using the housekeeping gene GAPDH. In each run, samples were tested in duplicate or triplicate, and repeated as indicated in the Results.

### Western blotting

Western blotting was performed as previously described [14] with modifications. Briefly, the cells were washed twice with cold PBS, harvested in cell lysis buffer (Cell Signaling Technologies) with protease and phosphatase inhibitors, and incubated for 20’ on ice. After centrifugation at 13,000 rpm × 10’ at 4°C, supernatants were aliquotted and stored at −80°C. Based upon the BCA assay (Thermo Scientific), 20–25 μg of protein from each sample in LDS loading buffer with reducing agent (Life Technologies) was denatured at 70°C × 10’, separated by electrophoresis using 10% Bis-Tris Plus gels (Life Technologies) and transferred to a nitrocellulose membrane using the iBlot Dry Transfer System (Life Technologies). Membranes were blocked in 1X PBS/Tween-20 (PBST) with 5% milk × 2 hrs at room temperature, then incubated overnight at 4°C with primary antibodies at 1 μg/ml in 1X PBST, washed and incubated for 2 hrs with GAR-HRP or GAM-HRP at room temperature. Protein bands were visualized using Chemiluminescent Substrate kit (Thermo Scientific) and the ChemiDoc MP imaging system (Bio-Rad). After probing for pERK1/2, membranes were incubated with stripping buffer (Thermo Scientific) and re-probed with anti-ERK1/2, anti-c-FOS, or anti-GAPDH primary Abs, as indicated, followed by appropriate secondary HRP-conjugated Abs and substrate. Band intensity of phosphorylated proteins was normalized as indicated in Results using Image Lab software and GIMP software (Bio-Rad). Data are representative of 2–5 experiments for each cell line, with duplicates in each experiment.

### Human specimens & immunohistochemistry (IHC)

The use of archival specimens, protocol #964456, was approved by the AU IRB. The sections from 17 randomly selected OSCC cases of the tongue (*n* = 10) and gingivae (*n* = 7) were reviewed by Board-certified oral and maxillofacial pathologist (ZBK) to verify uniform application of diagnostic criteria. Standard single-color IHC was performed as described previously [14, 59]. Briefly, 4 μm sections of formalin-fixed, paraffin-embedded archival tissue were deparaffinized and rehydrated by standard pathology laboratory methods. Heat-induced epitope retrieval was performed in a 97°C water bath for 20’ in citrate buffer, pH 6.0 (Thermo Scientific) and the sections were re-equilibrated in PBS. Endogenous peroxidase and non-specific antibody binding were blocked (Ultra-V block and Hydrogen Peroxide, LP Value kit, Thermo Fisher), and then sections were incubated at 4°C overnight with 2 μg/ml polyclonal rabbit Ab anti-TLR2 (#2229, Cell Signaling Technology) or anti-A2a (AVIVA Systems Biology). Negative controls included non-specific polyclonal rabbit Ab at 2 μg/ml (Abcam, Cambidge MA, USA). Primary Ab binding was detected using Enhancer, HRP-Polymer, and diaminobenzidine tetrahydrochloride (DAB) substrate, all according to the manufacturer's instructions (Thermo Fisher). All incubations, except the non-specific protein binding block, were separated by washing with PBS. Coverslips were applied with aqueous mounting medium (Thermo Fisher) and photomicrographs were obtained (Olympus Corp., Tokyo, Japan).

### BrdU incorporation assay

Cells were seeded at 1000 cells/well in 96 well plate and allowed to attach and equilibrate. The following day cells were pre-treated with 1 μM ERK inhibitor U0126 for 30’ as indicated, then incubated for 72 hrs with or without the indicated stimuli, as described in Experimental Set-up. Cell proliferation was measured using the BrdU Cell Proliferation Assay kit (Cell Signaling Technologies) and performed after incubation with BrdU for 24 hrs according to the manufacturer's instructions. Briefly, after fixing/denaturing the DNA, mouse monoclonal anti-BrdU Ab was added to the plates for 30’ at room temperature, followed by washing and incubating with HRP-labeled secondary Ab for 30’. Washed plates were then developed with 3,3′,5,5′-Tetramethylbenzidine (TMB) substrate. Absorbance at 450 nm was measured using the Epoch Microplate Spectrophotometer (BioTek Instruments Inc., Winooski, VT, USA). Relative values of treated vs untreated cells were calculated.

### Caspase-3 activity assay

Cells were plated at 150,000 cells/well in 6-well plates. After attachment and equilibration, cells were treated, as indicated, with ERK inhibitor U0126 for 30’, then incubated for 24 hrs with or without the indicated stimuli as described in the Experimental Set-up. Cells were harvested and caspase-3 activity was measured using the fluorogenic substrate N-Acetyl-Asp-Glu-Val-Asp-7-amido-4-trifluoromethylcoumarin (Ac-DEVD-AFC, Sigma-Aldrich). After harvesting, cells were lysed in 10 mM Tris-HCl, 10 mM NaH2PO4/NaHPO4 (pH 7.5), 130 mM NaCl, 1% Triton-X-100, and 10 mM Na4P2O7 and then incubated with 20 mM Hepes (pH 7.5), 10% glycerol, 2 mM DTT, and 25 μg/ml Ac-DEVD-AMC at 37°C for 2 h. The release of AFC was analyzed by the BioTek Synergy H1 Microplate Reader (BioTek Instruments Inc.) using excitation/emission wavelengths 390/510 nm. Relative caspase-3 activity values of stimulated vs. untreated cells were calculated.

### Statistical analysis

Statistical significance of differences between groups was assessed by one-way ANOVA, including Tukey-Kramer post-tests for multiple comparisons. All analyses were performed using the Prism software (GraphPad, San Diego, CA, USA). Probability values (P) of <0.05 were considered indicative of significant differences between data sets.

## SUPPLEMENTARY MATERIALS FIGURE AND TABLE



## Abbreviations

ADP: adenosine diphosphate
AFC: amido-4-trifluoromethylcoumarin
AMP: adenosine monophosphate
ANOVA: analysis of variance
AP-1: activator protein-1
AR: adenosine receptor
ATP: adenosine triphosphate
BCA: bicinchonionic acid
BrdU: bromodeoxyuridine
BSA: bovine serum albumin
CCL2: C-C motif chemokine ligand 2
CD: cluster of differentiation
DAB: diaminobezidine
DC: dendritic cell
DMEM: Dulbecco's Modified Eagle Medium
DMSO: dimethyl sulfoxide
E-NTPDase1: ecto-nucleoside triphosphate diphosphohydrolase 1
Ecto5’NTase: ecto-5′-nucleotidase
ED: epithelial dysplasia
EGF: epidermal growth factor
EGFR: epidermal growth factor receptor
ERK: extracellular signal regulated kinase
FBS: fetal bovine serum
G^pos^: Gram-positive
G^neg^: Gram-negative
GAM: goat-anti-mouse
GAPDH: glyceraldehyde 3-phosphate dehydrogenase
GAR: goat-anti-rabbit
HBSS: Hanks balanced salt solution
HRP: horseradish peroxidase
Hrs: hours
IRAK: IL-1 receptor-associated kinase
KSFM: Keratinocyte Serum-Free medium
LPS: lipopolysaccharide
MAPK: mitogen-activated protein kinase
MyD88: myeloid differentiation primary response gene 88
NECA: 5′-N-Ethylcarboxamidoadenosine
NF-kB: nuclear factor kappa-light-chain-enhancer of activated B cells
OSCC: oral squamous cell carcinoma
PBS: phosphate buffered saline
PBST: phosphate buffered saline/Tween 20
PGE_2_: prostaglandin E_2;_ PMSF: phenylmethylsulfonyl fluoride
qRT-PCR: quantitative real-time polymerase chain reaction
STAT: signal transducer and activator of transcription
TLR: toll-like receptor
TMB: 3,3′,5,5′-Tetramethylbenzidine
TNF: tumor necrosis factor
VEGF: vascular endothelial growth factor
ND: not done
SD: standard deviation.
