# Supplementary material for: Toll-like receptor 2 induces adenosine receptor A2a and promotes human squamous carcinoma cell growth via extracellular signal regulated kinases ½
Source: Oncotarget. 2017 Dec 30;9(6):6814–29. doi: 10.18632/oncotarget.23784 (PMC5805517; doi:10.18632/oncotarget.23784)
Supplement: Supplementary file 1 [file oncotarget-09-6814-s001.pdf]

Toll-like receptor 2 induces adenosine receptor A2a and promotes human squamous carcinoma cell growth via extracellular signal regulated kinases ½

SUPPLEMENTARY MATERIALS

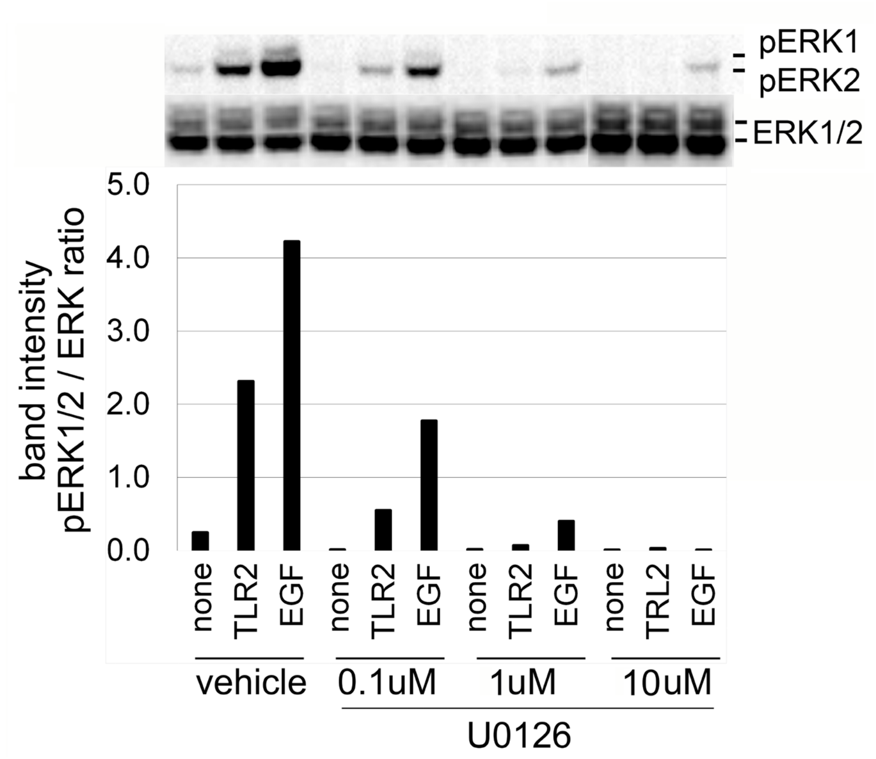

Supplementary Figure 1: Titration of ERK1/2 inhibitor U0126. Experimental set-up is described in the Materials and Methods. Briefly, PCI13 OSCC cells were plated, equilibrated overnight, and then incubated alone or with ERK inhibitor for 30 minutes at 0.1–10 µM (as indicated), followed by adding TLR2/1+TLR2/6 stimuli or EGF. The 1 µM dose was considered sufficient and selected for the experiments shown in Figure 4.

Supplementary Table 1: (**A**) Relative mRNA expression for AR and TLR in unstimulated cells. A1, A2b and A3 AR mRNA expression normalized to GAPDH was compared to A2a AR. (**B**) Levels of TLR1, TLR4 and TLR6 mRNA normalized to GAPDH were compared to those of TLR2 in unstimulated cells

**(A) Average Background AR mRNA Expression Relative to A2a**				
Cell lines	**A1**	**A2a**	**A2b**	**A3**
**THP1**	93%	100%	148%	172%
**keratinocytes**	none	100%	208%	none
**PCI13**	none	100%	205%	none
**Cal27**	none	100%	222%	none
**FaDu**	none	100%	199%	none
**SCC4**	none	100%	183%	none
**UMSCC1**	none	100%	181%	none
**UMSCC19**	none	100%	172%	none

**(B) Average Background TLR mRNA Expression Relative to TLR2**				
**Cell lines**	**TLR1**	**TLR2**	**TLR4**	**TLR6**
**THP1**	49%	100%	57%	47%
**keratinocytes**	63%	100%	108%	78%
**PCI13**	66%	100%	83%	81%
**Cal27**	66%	100%	84%	80%
**FaDu**	nd	100%	nd	nd
**SCC4**	nd	100%	nd	nd
**UMSCC1**	nd	100%	nd	nd
**UMSCC19**	83%	100%	120%	135%
